# Health Equity and Human Papillomavirus Vaccine Interventions for Adolescents: A Systematic Review

**DOI:** 10.3390/vaccines13050485

**Published:** 2025-04-30

**Authors:** Sarah B. Maness, Lois Coleman Carpenter, Idara Akpan, Nubwa St. James, Daniela Romero-Cely, G. J. Corey Harmon, Miranda Cano, Erika L. Thompson

**Affiliations:** 1 Department of Health Education and Promotion, East Carolina University, Greenville, NC 27858, USA; 2 Department of Health Promotion Sciences, University of Oklahoma Health Sciences Center, Oklahoma City, OK 73104, USA; lois-carpenter@ouhsc.edu (L.C.C.); nubwa-stjames@ouhsc.edu (N.S.J.); 3 Population & Community Health, University of North Texas Health Science Center, Fort Worth, TX 76104, USA; idaraakpan@my.unthsc.edu; 4 Brody School of Medicine, East Carolina University, Greenville, NC 27834, USA; romerocelyd22@students.ecu.edu; 5 Laupus Health Sciences Library, East Carolina University, Greenville, NC 27834, USA; harmong18@ecu.edu; 6 Department of Quantitative and Qualitative Health Sciences, UT Health San Antonio, San Antonio, TX 78229, USA; canom4@uthscsa.edu (M.C.); thompsone1@uthscsa.edu (E.L.T.)

**Keywords:** human papillomavirus, health equity, systematic review

## Abstract

Background/Objectives: Human papillomavirus (HPV) causes multiple types of cancer, and demographic-based inequities in HPV-related cancers persist. Behavioral interventions have increased HPV vaccination uptake, yet it is unclear how intervention effects vary by demographics. The purpose of this study was to examine whether existing HPV vaccine interventions for adolescents have unequal effects on HPV vaccine uptake. Methods: We searched MEDLINE via PubMed, PsycINFO, CINAHL, Scopus, and Cochrane CENTRAL in October 2023. The search strategy combined keywords and subject terms for HPV vaccine, interventions/health promotion, and adolescents. Studies were included in final analyses if they were peer-reviewed, published in the US between 2006 and 2023, included outcome measures from an evidence-based HPV vaccination intervention, included adolescents aged 9–17, and demographic variables for age, race/ethnicity, income/SES, or geographic region. Studies were excluded if they were review articles, abstract-only, dissertations or theses, non-English language, non-US-based, or outside the age range of 9–17. Studies were also excluded if they did not include an intervention, outcome evaluation measures, or demographic measures. The screening and extraction processes were independently performed by multiple reviewers using Covidence software. Results: Ultimately, 74 articles were included for full extraction. Sex was the most common demographic variable analyzed by the HPV vaccine (n = 38), followed by race/ethnicity (n = 15), income/SES (n = 6), and geographic region (n = 6). Conclusions: Few interventions assess whether intervention results differ by demographics, making it unclear whether these interventions reduce health inequities. This review included a wide variation in study designs, limiting our ability to uniformly assess study conclusions.

## 1. Introduction

Human papillomavirus (HPV) causes six types of cancer—anal, cervical, oropharyngeal, penile, vaginal, and vulvar [1]—and is responsible for nearly 38,000 new cancer cases per year [2]. Among those diagnosed with HPV-related cancers, cervical cancer is the most common in women, while oropharyngeal cancer is the most common HPV-related cancer in men. Certain populations are disproportionately affected by these cancers. For example, American Indian/Alaskan Native, Hispanic, and Black women have higher cervical cancer incidence rates compared to white women, while cervical cancer mortality is higher among Native Hawaiian or Other Pacific Islander women [3]. Similarly, mortality rates due to oropharyngeal cancer are higher among Native Hawaiian or Other Pacific Islander men and women. Furthermore, women in rural areas experience higher cervical cancer incidence rates, and both men and women in rural areas have higher oropharyngeal cancer incidence rates compared to those in urban areas [4].

Although the HPV vaccine can prevent most of these cancers, uptake remains a challenge. HPV vaccination is recommended for 11–12-year-olds and can be administered as early as 9 [5]. However, as of 2022, only 58.6% of 13–15-year-olds in the United States were up-to-date on the HPV vaccination series [6], which is well below the national target of 80% [7]. Disparities in vaccination rates are particularly evident among uninsured children and children living in non-metropolitan areas, who have lower rates of vaccine uptake and are less likely to complete the HPV vaccine series [8]. These disparities are likely to have long-term implications, further increasing HPV-related cancer incidence and mortality rates among affected populations.

Over the past two decades, behavioral interventions have been a primary strategy to increase HPV vaccination uptake and completion. The 2018 President’s Cancer Panel report emphasized the importance of provider recommendations, clinical system changes, and communication campaigns as critical strategies to accelerate HPV vaccination uptake [9]. A recent systematic review identified several modifiable factors at the individual, provider, and clinic levels that influence vaccination outcomes among adolescents [10]. The authors developed a multilevel framework illustrating how these factors interact and can be targeted to improve vaccination rates. While this framework offers a comprehensive approach to intervention development, it highlights the need for further research to understand how intervention effects may differ across health disparity populations. Avni-Singer et al. [11] conducted a systematic review of HPV vaccine impact studies (i.e., change in population-level burden of disease) to examine how extensively researchers include racial, ethnic, and socioeconomic characteristics to assess for disparities. However, only two studies out of 23 stratified the results by sociodemographic characteristics, which limits our ability to understand whether prevention strategies are reaching populations with the largest burden of disease. Thus, if we are to consider the pathway to HPV vaccine uptake through the lens of behavioral interventions, we must also interrogate the diversity and inclusivity of samples used to establish evidence for these interventions.

Given that HPV vaccination among adolescents in the United States continues to be suboptimal, as well as the presence of health disparities for HPV vaccination and HPV-related cancers, we must investigate the quality and equity of the evidence for HPV vaccine behavioral interventions. By prioritizing certain strategies for HPV vaccination, there is the potential risk of perpetuating and/or creating health disparities if the evidence generated is based on homogeneous samples. Thus, the purpose of this study was to examine whether existing HPV vaccine interventions for adolescents have unequal effects on HPV vaccine uptake.

## 2. Methods

### 2.1. Overview

Our systematic review of HPV vaccination interventions required a focus on evidence-based interventions. Thus, with guidance from the National HPV Vaccination Roundtable’s Best Practices Learning Collaborative [12], we assembled a list of interventions to include within our systematic review (Table 1). This systematic review was registered with Open Science Framework [13] and followed PRISMA guidelines [14].

### 2.2. Systematic Search

Adhering to the guidelines described by the Cochrane Handbook for Systematic Reviews of Interventions, we iteratively developed a search for use in the following databases: MEDLINE via PubMed (using the advanced search), PsycINFO, the Cumulative Index to Nursing and Allied Health Literature (CINAHL), Complete, Scopus, and Cochrane CENTRAL (searched via Ovid interface). No filters or limits were applied to any of the searches. The initial search was run on 27 September 2023. One of the authors (CH), who is a health sciences librarian, developed the search strategy by combining keywords and subject terms for the main concept domains: HPV vaccine, interventions/health promotion, and adolescents. We iteratively developed a comprehensive list of search terms then developed a search string in PubMed. The PubMed search was peer-reviewed by a second research librarian using the Peer Review of Electronic Search Strategies, or PRESS checklist before it was translated to the other four databases. The full search string can be found in Appendix A.

### 2.3. Selection Criteria

Studies were included in the final analyses if they met the following criteria:

Peer-reviewed, published in the US between January 2006 and September 2023, include outcome measures from an evidence-based HPV vaccination intervention. Evidence-based HPV vaccination intervention was determined by inclusion on the HPV Round Table: HPV Vaccination Best Practices Learning Collaborative. The interventions had to focus on HPV vaccination among adolescents 9–17 and may include system-level, provider, and parental-focused activities. Demographics had to include HPV vaccination initiation by race, ethnicity, sex, gender, and/or region.

Exclusion criteria were studies without primary data (review articles), non-English language, non-US-based, focused on HPV vaccination outside the age range of 9–17, and did not include evidence-based intervention, outcome evaluation measures, or demographic measures. Additional exclusions were if the article was abstract only, a dissertation, or a thesis.

### 2.4. Screening and Eligibility

Using EndNote Citation Manager (version 20) and Covidence Systematic Review software (covidence.org), duplicates were removed. After that, using the latter software, study titles, and abstracts from the searches were screened against the eligibility criteria. Following that, the qualified studies were screened for full-text review, recording the reasons for which studies were subsequently excluded. The screening process was independently performed by multiple reviewers (IA, SBM, ET, LC, NSJ, SA, DRC). Conflicts were resolved by assessment from a third reviewer, a senior member of the research team (SBM, ET, LC).

### 2.5. Data Extraction

**Figure 1:**
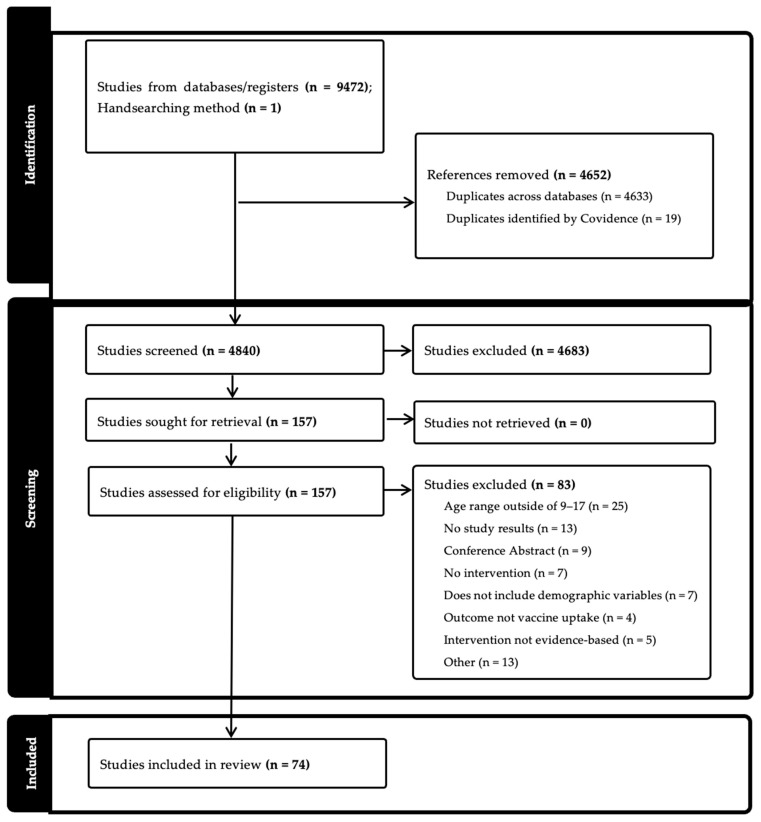
PRISMA Flowchart [14].

After the screening process, data were extracted in the following areas: general information (title; lead author; year of publication), characteristics of included studies (aim of study; setting; start and end date; study design; population description; inclusion and exclusion criteria; method of recruitment; description of intervention; type of intervention; measurement of race/ethnicity; sex; income/socioeconomic status; geographic region; other demographics; main HPV outcome variable), and outcomes (number of participants; change in HPV vaccine uptake measurement and statistics; stratification by sex, race, income/socioeconomic status, geographic region, other demographics; limitations; main conclusions). We assessed the risk of bias, including pre-post intervention data, control or comparison group, and random assignment (Figure 1).

Data from the study were independently extracted by two team members (IA, SBM, ET, LC, NSJ, SA, DRC) using Covidence software [15].

## 3. Results

### 3.1. Summary of Findings

A total of 9472 results were found across all five databases (CINAHL 1144; PsycINFO 280; PubMed 3542; Scopus 4050; Cochrane CENTRAL 456), and 4633 duplicates were removed. An additional 1 article was identified through handsearching. After entering into Covidence, 19 additional articles were moved. A total of 4840 items were included in the title and abstract screening. After initial screening, team members completed a full-text review of 157 titles. Ultimately, 74 articles were included for full extraction. (Table 2). The studies reviewed measured either HPV vaccine initiation (n = 16), completion (n = 8), both (n = 44), or other (n = 6) (e.g., whether the visit included a vaccine or not). Study settings were primarily in clinics (n = 52) but also included community (n = 8), school (n = 5), online (n = 1), other (n = 1), or multiple settings (n = 5).

### 3.2. Methodology

Less than half of the included studies used an experimental design (n = 33) (Table 2). Sixteen studies were quasi-experimental, and the remaining studies used a non-experimental design (n = 25). Sample sizes for adolescents ranged from 39 to 312,227. Clinic studies ranged from 22 clinics to 267 clinics. Samples included a focus on adolescents (31 studies), adolescents and parents (17 studies), parents (10 studies), and other combinations of parents, adolescents, and/or providers (16 studies). Examining each study by National HPV Vaccination Roundtable: HPV Vaccination Best Practices Learning Collaborative intervention type [12], 51 studies were multi-component (i.e., had more than one intervention type), while 23 studies only used one component. Studies that focused on only one intervention type were most commonly Patient Education (n = 9), followed by Patient Outreach (n = 8), Provider Education (n = 5), and having an EHR feature (n = 1). Among studies that combined intervention types, combinations ranged from two to all six types of interventions. Among the intervention types, Patient Education was the most common with 46 studies, followed by Provider Education and Patient Outreach (38 studies), EHR feature (25), Standing Orders (11), Patient Scheduling (10), and Provider Incentives (5). Fifty studies analyzed HPV vaccine uptake by at least one demographic variable. Information about specific demographic variables is described in the sections below.

### 3.3. Sex

Thirty-nine studies analyzed HPV vaccine uptake by sex. All 39 studies measured sex as a category of either male or female. Of these studies, 27 examined between-group differences, and 9 found significant differences by sex [27,36,49,63,66,81]. Only one study found that males were more likely to initiate HPV vaccination than females [30]; the rest found females have greater vaccine uptake (Table 3).

### 3.4. Race/Ethnicity

Fifteen studies measured race/ethnicity and HPV vaccine uptake. Of these studies, 12 examined between-group analyses by race/ethnicity and differences in HPV vaccine uptake. Five studies found at least one significant association between a variable of race/ethnicity and HPV vaccine uptake or completion (Table 3). Rickert et al. [69] found that Hispanic participants were more likely than non-Hispanic participants to initiate a first dose. Results from Caskey et al. [30] indicated that Hispanic, White, and Other race/ethnicity participants were more likely to initiate, receive two doses, or complete the HPV vaccination series than Black participants. Additional studies found that non-White adolescents were more likely to initiate and complete the HPV vaccine series than White participants [21,36,63,88].

### 3.5. Income/Socioeconomic Status

Five studies analyzed between-group differences by income or socioeconomic status and HPV vaccination. Using poisson regression to estimate relative risk, one study found that when adjusting analyses for household income, unvaccinated daughters had a higher likelihood of vaccine initiation (RR = 2.6, 95% CI:1.4–4.9) and completion (RR = 4.0, 95% CI: 1.2–13.1) in the intervention versus control participants [86] (Table 3). Another study using prevalence ratios found lower vaccine uptake among households earning less than USD 75,000 per year (PR = 6.33, 95% CI 5.51–7.26) and whose mothers had less than a high school education (PR = 3.91, 95% CI 3.05–5.02) [16]. The remaining studies did not have substantial differences in uptake by income or socioeconomic status [68,71,78].

### 3.6. Geographic Region

Seven studies analyzed geographic regions and HPV vaccine uptake. Among these studies, geography was defined by either urban/suburban area, zip code, rurality, state, or region. Four of the studies statistically compared group differences in HPV vaccine uptake based on geographic region. In two studies, no significant differences were found between Southwestern and Northwestern States [46] or based on rural and urban areas of upstate New York [78]. One found significantly more immunization opportunities in urban versus suburban clinics [57], and another found prevalence ratios indicating that participants in the US South (PR = 6.01, 95% CI 5.38–6.72) and West (PR = 5.67, 95% CI 4.56–7.05) were significantly less likely to complete HPV vaccination [16] (Table 3).

### 3.7. Other Demographic Variables

Multiple studies reported at least one other demographic variable in relation to HPV vaccine uptake. Of these studies, the most common additional demographics were analyzing age by subgroups (n = 26) and Insurance status (n = 12). Parental information was also recorded in multiple studies, including parental education (n = 3), parental marital status (n = 2), and parental age (n = 2). Two additional studies measured HPV vaccination uptake in the English language [63,68] (Table 3).

## 4. Discussion

This systematic review found that although a multitude of evidence-based interventions exist to increase HPV vaccination, few stratify vaccine uptake by multiple demographic variables. For each demographic assessed, at least one study found significant differences in HPV vaccine uptake or completion. The demographics most commonly represented among the intervention studies were age group and sex. Since our study only assessed a limited age range (ages 9–17), information stratified by age group only included comparing older versus younger adolescents. We found that 27 studies stratified outcome variables by sex, more than by any other demographic category. Recent data from NIS-Teen indicates sex differences persist in HPV vaccination, and in 2023, 42.9% of girls aged 9–17 had received one or more HPV vaccine doses in comparison with 34.6% of boys [90].

A recent systematic review focusing on area-level variation in HPV vaccination among adolescents and young adults in the US found that HPV vaccination was associated with area-level poverty, urbanicity/rurality, and racial/ethnic composition [91]. We know from previous research that these demographic-related inequities exist in HPV vaccine uptake, yet our findings indicate that evaluations of HPV vaccine interventions largely do not assess whether these disparities persist post-intervention [3,4].

Our findings indicate that when removing categorization by age groups, only around half of the studies assessed any other demographic variable. This limits the extent to which we can draw overall conclusions about how interventions may differentially increase vaccine uptake across different populations. Additionally, among studies that included demographic variables in analyses, some reported only pre- and post-intervention data rather than examining between-group differences. This methodological strategy limits the ability to assess whether interventions are more successful based on specific demographic characteristics.

Marked inequities in HPV vaccine uptake by race/ethnicity exist, yet only 16 of the 73 studies included in our review assessed outcomes based on race/ethnicity. Additionally, none of the studies specifically included Native Hawaiians or other Pacific Islanders despite higher rates of both cervical and oropharyngeal cancers among this group [4]. The specific measures of race/ethnicity differed by study, but overall, four studies found that White participants were less likely than those of other races/ethnicities to be vaccinated. This aligns with findings by race/ethnicity from the nationally representative NIS-Teen survey [8].

Research indicates that adolescents from disadvantaged areas, living in poverty, or otherwise socially vulnerable are less likely to initiate or complete HPV vaccination [92,93]. However, our review found only six studies that examined HPV vaccine intervention outcomes by income or socioeconomic status, with limited significant findings. This leaves us unable to examine whether evidence-based HPV vaccination interventions are differentially effective based on factors related to income. Similarly, previous studies have also found links between geography and HPV vaccination, including lower uptake in rural areas in comparison with urban areas [94]. This review found six studies that measured geographic region, and each was measured differently. Due to the limited number of studies and variation in measurement, we cannot draw conclusions about findings across these studies. Both demographic areas emphasize the need to be able to assess the effectiveness of HPV vaccination stratified by key demographic factors.

This study has many strengths, including that it was framework-based, and each study was extracted by multiple reviewers. However, this study is not without limitations. The review included study designs that were both experimental and non-experimental, limiting our ability to uniformly assess study conclusions. Additionally, this study was specific to adolescents, and findings cannot be extrapolated to other HPV-vaccine-eligible age groups. This systematic review also only included published studies, which may have excluded studies in which the findings were not significant or otherwise not published.

## 5. Conclusions

HPV vaccination uptake and HPV-related diseases differ based on demographic factors, yet interventions to improve vaccine uptake largely do not assess whether interventions are successful among populations differentially impacted. To achieve health equity in HPV vaccination, we must implement programs that improve vaccination uptake across demographic groups. Future evidence-based HPV vaccination uptake interventions should assess outcomes stratified by demographic variables, especially demographics with documented evidence of inequitable outcomes in HPV-related disease. This will help ensure HPV interventions are not only effective but also equitable, reaching populations most impacted by HPV-related diseases.

## Figures and Tables

**Table 1:** HPV Round Table Intervention Types and Descriptions [12].

HPV Round Table: HPV Vaccination Best Practices Learning Collaborative, Interventions	
Intervention Type and Descriptions	
Provider Education	Community meetings, pediatric provider meetings, guest speakers, weekly newsletters, encouraging articles, social media, and webinars.
Provider Incentives	Linking adolescent immunization to provider performance and compensation.
Patient Education	Incorporating patient education resources in exam rooms, flyers, letters, emails, texts, HER features, webinars, handouts, resources, and conversations.
Patient Outreach	Gap-closure and missed opportunity reports, personal phone calls, scripts to answer common questions, postcard campaigns, letters, calls, HER features such as reminder messages.
Patient Scheduling	Telehealth and virtual exam rooms, adding HPV vaccines to drive through flu vaccine settings, in-school onsite vaccine services, back-to-school campaigns, annual wellness visits, standing orders.
Electronic Health Record (EHR) Feature	Reports and alerting messages, overdue for visit reports, overdue for vaccination reports, alerts with real-time information during visits.

**Table 2:** Description of Studies Included in Review.

Author/ Year	Setting	Sample	Sample Size (Youth)	Study Design	Intervention Type	HPV Vaccine Outcome Variable	Demographics Analyzed by HPV Outcome Variable
Agana-Norman 2022 [16]	Other	Male youth	97,587	Non-experimental	Provider Education; Patient Education	Initiation and Completion	Race/ethnicity; Income/SES; Geography; Parental Education; Age
Aragones 2015 [17]	Community	Mexican/Mexican American parents	69	Quasi-experimental	Patient Education	Completion	Sex
Baldwin 2021 [18]	Clinic	Parents of pediatric patients	161	Experimental	Patient Education; Electronic Health Record (EHR) Feature	Vaccination status	-
Bastani 2022 [19]	Community	Youth Caregiver	225	Experimental	Patient Education	Initiation and Completion	-
Beck 2021 [20]	Clinic	Parents of youth 11–17	24	Non-experimental	Patient Education	Initiation	-
Berenson 2019 [21]	Clinic	Families with children	2162	Non-experimental	Provider Education; Patient Education; Patient Scheduling; EHR Feature	Initiation and Completion	Sex; Race/ethnicity; Age
Berenson 2020 [22]	Clinic	Youth 9–17	21,395	Quasi-experimental	Provider Education; Patient Education; Patient Scheduling	Whether visit included vaccination	Race/ethnicity; Age
Bernstein 2022 [23]	Clinic	Youth 11–12	128	Non-experimental	Provider Education; Patient Education; Patient Outreach; EHR Feature; Standing Orders	Initiation	-
Biehl 2023 [24]	Clinic	Youth 11–17	1374	Non-experimental	Provider Education; Patient Education; Patient Outreach; Standing Orders	Initiation and Completion	Sex; Insurance
Bonville 2019 [25]	Clinic	Youth 11–12; Providers	232	Non-experimental	Provider Education; Provider Incentives; EHR Feature	Initiation and Completion	-
Bowden 2017 [26]	Clinic	Youth 9–13	265	Non-experimental	Provider Education; Patient Education; EHR Feature	Initiation	-
Brewer 2017 [27]	Clinic	Youth 11–17	37,796	Experimental	Provider Education	Initiation and Completion	Sex; Insurance
Brodie 2018 [28]	Clinic	Youth 9–10	6703	Non-experimental	Provider Education; EHR Feature	Initiation	-
Buller 2021 [29]	Online	Mothers and daughters 14–17	469	Non-experimental	Patient Education	Initiation and Completion	-
Caskey 2017 [30]	Clinic	Youth 11–17; Parent/ guardian	188	Other: Quasi-randomized trial	Patient Outreach	Initiation and Completion	Sex; Race/ethnicity; Insurance
Cassidy 2014 [31]	Clinic	Parents of girls 11–12	53	Quasi-experimental	Provider Education; Patient Education; Patient Scheduling; EHR Feature	Initiation and Completion	-
Cates 2014 [32]	Clinic; Community; Online	Boys 11–13	25,870	Quasi-experimental	Provider Education; Patient Education	Initiation	Race/ethnicity; Age
Cates 2018 [33]	Clinic	Youth 9–14	147,294	Other: Quasi-experimental	Provider Education; Provider Incentives; Patient Education	Initiation and Completion	Sex; Age
Cates 2020 [34]	Clinic; Online	Youth 11–12; Parents	55	Experimental	Patient Education	Initiation and Completion	-
Coley 2018 [35]	Community	Youth 11–13; Parent/ guardian	303,965	Experimental	Patient Education; Patient Outreach	Initiation and Completion	Sex
Cox 2022 [36]	Clinic	Youth 9–13	12,270	Non-experimental	Provider Education; Patient Education; Patient Outreach; Patient Scheduling; EHR Feature; Standing Orders	Initiation and Completion	Sex; Race/ethnicity; Age
Daley 2014 [37]	School	Girls 6–8th grade	-	Experimental	Patient Outreach	Initiation and Completion	-
Dang 2023 [38]	Clinic	Youth 11–17	498	Non-experimental	Provider Education; Patient Education; Patient Outreach; EHR Feature; Standing Orders	Initiation and Completion	-
Davis 2022 [39]	Clinic	Youth 12	39	Non-experimental	Provider Education	HPV vaccination rate	Race/ethnicity; Insurance Status; Parental Age
Dempsey 2018 [40]	Clinic	Youth 11–17	43,132	Experimental	Provider Education; Patient Education	Initiation and Completion	Sex; Age
Dixon 2019 [41]	Clinic	Parents/guardians of youth 11–17	1596	Experimental	Patient Education; EHR Feature	Initiation and Completion	-
Eldred 2015 [42]	School	Middle school students	184	Quasi-experimental	Patient Education; Patient Outreach; Patient Scheduling	Initiation	Sex
Fiks 2013 [43]	Clinic	Girls 11–17	22,486	Experimental	Provider Education; Patient Education; Patient Outreach; Patient Scheduling; EHR Feature	Initiation and Completion	Age
Fisher-Borne 2018 [44]	Clinic	Youth 11–12	>20,000	Non-experimental	Provider Education; Patient Outreach; EHR Feature; Standing Orders	Initiation and Completion	-
Gilkey 2022 [45]	Clinic	Youth 11–17	176,189	Experimental	Provider Education; Provider Incentives; Standing Orders	Initiation and Completion	Geography; Age
Gilkey 2023 [46]	Clinic	Practices	312,227	Experimental	Provider Education	Initiation	Age
Glenn 2022 [47]	Clinic	Youth 11–17	14,738	Experimental	Provider Education; Patient Outreach; Standing Orders	Initiation and Completion	Sex
Glenn 2023 [48]	Clinic	Youth 12; Caregiver	877	Quasi-experimental	Patient Outreach; Patient Scheduling	Receipt of next-dose vaccination	Sex
Gurfinkel 2021 [49]	Clinic	Youth 11–14	69,286	Experimental	Patient Outreach	Initiation and Completion	Sex; Age
Henrikson 2018 [50]	Clinic	Parents of youth 10–12	1624	Experimental	Patient Education; Patient Outreach	Initiation and Completion	Sex; Age
Joseph 2016 [51]	Clinic	Mothers of daughters 11–15	200	Experimental	Patient Education	Initiation and Completion	-
Kaul 2019 [52]	School	6th-, 7th-, and 8th-grade students	2307	Quasi-experimental	Patient Education	Initiation	Sex; Age
Kempe 2016 [53]	Clinic	Youth 11–17	929	Experimental	Patient Outreach	Completion	Sex; Race/ethnicity; Age
Krantz 2018 [54]	Clinic	Youth 13–17; Providers	314	Non-experimental	Provider Education; Patient Scheduling; EHR Feature	Completion	Sex
Lennon 2019 [55]	Community	African American youth 13–17	118	Quasi-experimental	Patient Education; Patient Outreach	Completion	-
Mackey 2019 [56]	Clinic; Other	Youth 11–12	247	Non-experimental	Provider Education; Patient Education; EHR Feature	Initiation and Completion	Sex
Mayne 2014 [57]	Clinic	Girls 11–17	17,016	Experimental	Provider Education; Patient Education; Patient Outreach; EHR Feature	HPV receipt	Geography
McLean 2017 [58]	Clinic	Youth 11–17	24, 658	Quasi-experimental	Provider Education; Patient Outreach	Initiation and Completion	-
O’Leary 2023 [59]	Clinic	Youth 9–13	25,888	Non-experimental	Provider Education; EHR Feature	Initiation and Completion	-
Parra-Medina 2015 [60]	Community	Hispanic women with daughters 11–17	372	Quasi-experimental	Patient Education; Patient Outreach	Initiation and Completion	Parental Employment; Marital Status; Education; Insurance
Paskett 2016 [61]	Clinic; Other	Parent/guardian of girls 9–16	337	Experimental	Provider Education; Patient Education	Initiation	-
Perkins 2020 [62]	Clinic	Youth 9–17; Parent/ guardian; Provider	16,136	Experimental	Provider Education	Initiation and Completion	Sex; Race/ethnicity; Geography; English Language
Perkins 2021 [63]	Clinic	Youth 13; Providers	3283	Non-experimental	Provider Education; Provider Incentives; Patient Outreach; EHR Feature; Standing Orders	Initiation and Completion	-
Potts 2019 [64]	Clinic	Parents of children 9–17	46	Non-experimental	Patient Education	HPV vaccination rate.	Sex
Rand 2015 [65]	Clinic	Youth 11–16; Parents	3812	Experimental	Patient Outreach; Patient Scheduling	Initiation and Completion	Sex; Age
Rand 2017 [66]	Clinic	Youth 11–17; Parents	749	Experimental	Patient Outreach	Completion	Sex
Rand 2018 [67]	Clinic	Youth 11–17	43,435	Non-experimental	Provider Education; Provider Incentives; EHR Feature; Standing Orders	Initiation and Completion	Sex
Richman 2019 [68]	Clinic	Parents of youth 9–17	257	Experimental	Patient Education; Patient Outreach	Competition	Sex; Race/ethnicity; Income/SES; English Language
Rickert 2015 [69]	School; Clinic; Other	Parents of youth 11–15	445	Experimental	Patient Education; Patient Outreach	Initiation and Completion	(All Parental) Sex; Race/ethnicity; Marital Status; Education; Insurance; Age
Santa Maria 2021 [70]	Community	Parents of youth 11–14	508	Experimental	Patient Education; Patient Outreach; Patient Scheduling	Initiation and Completion	Sex
Scarinci 2020 [71]	Community	Latina immigrants with daughters 9–12	278	Experimental	Patient Education; Patient Outreach	Completion	Income/SES
Shegog 2022 [72]	Clinic	Youth 11–17; Parents	375	Experimental	Patient Education; Patient Outreach; EHR Feature	Initiation	-
Smajlovic 2023 [73]	Clinic	Youth 13; Parents	81,000	Non-experimental	Provider Education; Patient Education; EHR Feature	Completion	Sex; Race/ethnicity
Staras 2015 [74]	Clinic	Youth 11–17; Parents	6123	Quasi-experimental	Provider Education; Patient Education; Patient Outreach	Initiation	Sex
Staras 2020 [75]	Other	Girls 11–17	2773	Quasi-experimental	Patient Outreach	Initiation	-
Steiner 2021 [76]	Clinic	Youth 11–14; Registered nurses	209	Quasi-experimental	Provider Education; Patient Education; EHR Feature; Standing Orders	HPV Vaccination	Sex
Strasel 2023 [77]	Clinic	Youth 9–10	367	Non-experimental	Provider Education; Patient Education; Patient Outreach; EHR Feature	Initiation	-
Szilagyi 2013 [78]	Clinic	Youth 10.5–17	4115	Experimental	Patient Outreach	Initiation and Completion	Sex; Income/SES; Insurance; Geography; Age
Szilagyi 2020 [79]	Clinic	Youth 11–19	62,118	Experimental	Patient Outreach	Initiation and Completion	Sex; Geography; Age
Szilagyi 2021 [80]	Clinic	Youth 11–17; Providers	104,438	Experimental	Provider Education	Initiation and Completion	Sex; Age
Underwood 2016 [81]	School	Girls under 18; Parent/guardian	360	Experimental	Patient Education; Patient Outreach	Initiation	Sex; Race/ethnicity; Age
Varman 2018 [82]	Clinic	Youth 9–17; Parent/ guardian; Providers	3393	Non-experimental	Provider Education; Patient Education; Patient Outreach	Initiation and Completion	Sex; Geography; Insurance
Vinci 2022 [83]	Clinic	Youth 11–16	8960	Non-experimental	Provider Education; Patient Outreach; EHR Feature	Initiation and Completion	Sex; Age
White 2022 [84]	School	7th grade students; Parents/guardians	1686	Quasi-experimental	Patient Education; Patient Outreach	Initiation	Sex; Race/ethnicity; Insurance
Wilkinson 2019 [85]	Clinic	Youth 11–17; Providers	1285	Experimental	EHR Feature	Initiation	Sex; Age
Winer 2016 [86]	Community	Mothers or legal guardians of girls 9–12	97	Experimental	Patient Education	Initiation and Completion	Income/SES; Age
Woodall 2021 [87]	Clinic	Parents and daughters 11–14	82	Experimental	Patient Education; Patient Outreach	Initiation and Completion	-
Zimmerman 2017 [88]	Clinic	Youth 11–17	9473	Non-experimental	Provider Education; Patient Outreach; Standing Orders	Initiation and Completion	Sex; Race/ethnicity; Insurance; Age
Zorn 2023 [89]	Clinic	Youth 11–17	45,859 in May 2022	Non-experimental	Provider Education; Patient Education; EHR Feature	Initiation and Completion	-

- indicates that study did not examine between-group differences for this demographic variable.

**Table 3:** Findings of studies that examined between-group differences for at least one demographic variable by HPV vaccine uptake.

Author/Year *	Sex	Age	Race/ Ethnicity	Income/ SES	Geography	Insurance	Other
Agana-Norman 2022 [16]	-	S	S	S	S	-	S
Aragones 2015 [17]	NS	-	-	-	-	-	-
Berenson 2019 [21]	NS	S	S	-	-	-	-
Berenson 2020 [22]	-	S	S	-	-	-	-
Biehl 2023 [24]	NS	-	-	-	-	NS	-
Brewer 2017 [27]	S						
Caskey 2017 [30]	S	-	S	-	-	NS	-
Cates 2014 [32]	-	S	NS	-	-	-	-
Cates 2018 [34]	S	S	-	-	-	-	-
Cox 2022 [36]	S	-	S	-	-	-	-
Davis 2022 [39]	-	NS	NS	-	-	NS	NS
Dempsey 2018 [40]	NS	-	-	-	-	-	-
Gilkey 2022 [45]	-	-	-	-	NS	-	-
Glenn 2022 [47]	S	-	-	-	-	-	-
Gurfinkel 2021 [49]	S	S	-	-	-	-	-
Henrikson 2018 [50]	-	NS	-	-	-	-	-
Kaul 2019 [52]	NS	S	-	-	-	-	-
Kempe 2016 [53]	NS	S	NS	-	-	-	-
Mayne 2014 [57]	-	-	-	-	S	-	-
Parra-Medina 2015 [60]	-	S	-	-	-	S	S
Perkins 2020 [62]	S	-	S	-	-	S	S
Rand 2015 [65]	NS	NS	-	-	-	-	-
Rand 2017 [66]	S	-	-	-	-	-	-
Richman 2019 [68]	NS	NS	NS	NS	-	-	NS
Rickert 2015 [69]	NS	S	S	-	-	NS	NS
Santa Maria 2021 [70]	NS	-	-	-	-	-	-
Scarinci 2020 [71]	-	-	-	NS	-	NS	NS
Szilagyi 2013 [78]	NS	NS	-	NS	-	NS	-
Szilagyi 2020 [79]	NS	NS	-	-	NS	-	-
Szilagyi 2021 [80]	NS	NS	-	-	-	-	-
Underwood 2016 [81]	S	NS	NS	-	-	-	-
Varman 2018 [82]	-	-	-	-	S	S	-
Vinci 2022 [83]	NS	S	-	-	-	-	-
Wilkinson 2019 [85]	NS	NS	-	-	-	-	-
Winer 2016 [86]	-	S	-	S	-	-	-
Zimmerman 2017 [88]	S	S	S	-	-	S	-

* See Table 1 for study-specific measurement of vaccine uptake. S indicates a significant between-group difference by demographic and HPV vaccine uptake. NS indicates a non-significant finding. - indicates that study did not examine between-group differences for this demographic variable.

## Data Availability

Extracted data and template data collection form may be available based upon reasonable request of the corresponding author and approval of the research team.

## Appendix A. Full Search String for Systematic Review

((“Papillomavirus Vaccines”[Mesh] OR “HPV vaccine”[tiab] OR “HPV vac-cines”[tiab] OR “HPV vaccination”[tiab] OR “HPV vaccinations”[tiab] OR “Papillo-mavirus Vaccination”[tiab] OR “Papillomavirus Vaccinations”[tiab] OR “Human Papilloma Virus vaccination”[tiab] OR “Human Papilloma Virus Vaccinations”[tiab] OR “Papillomavirus Vaccine”[tiab] OR “Papillomavirus Vaccines”[tiab] OR “Human Papilloma Virus Vaccine”[tiab] OR “Human Papilloma Virus Vaccines”[tiab] OR “Human Papillomavirus Vaccine”[tiab] OR ((“Human Papillomavirus Viruses"[Mesh] OR "Papillomavirus Infections"[Mesh] OR “Human Papillomavirus Virus”[tiab] OR “Human Papillomavirus Viruses”[tiab] OR “Human Papillomavirus”[tiab] OR “Hu-man Papillomaviruses”[tiab] OR “Human Papilloma Virus”[tiab] OR “Human Papil-loma Viruses”[tiab] OR “HPV Human Papillomavirus”[tiab] OR “HPV Human Papil-lomaviruses”[tiab] OR HPV[tiab] OR “Papillomavirus Infection”[tiab] OR “Papillo-mavirus Infections”[tiab] OR “Human Papillomavirus Infection”[tiab] OR “Human Papillomavirus Infections”[tiab] AND ("Vaccines"[Mesh] OR "Vaccination"[Mesh] OR "Vaccination Refusal"[Mesh] OR vaccin*[tiab] OR preventive[tiab] OR preven-tion[tiab] OR immunization*[tiab] OR innoculat*[tiab]))) AND (“Patient Education as Topic”[Mesh] OR “Health Promotion”[Mesh] OR “Health Knowledge, Attitudes, Prac-tice”[Mesh] OR “Patient Acceptance of Health Care”[Mesh] OR Intervention[tiab] OR Interventions[tiab] OR “Patient Education”[tiab] OR “Education of Patients”[tiab] OR “Promotion of Health”[tiab] OR “Health Promotion”[tiab] OR “Health Promo-tions”[tiab] OR “Wellness Programs”[tiab] OR “Wellness Program”[tiab] OR “Health Campaigns”[tiab] OR “Health Campaign”[tiab] OR “Health knowledge”[tiab] OR “Health Attitude”[tiab] OR “Health Attitudes”[tiab] OR “Health Care Utiliza-tion”[tiab] OR “Patient Acceptance of Healthcare”[tiab] OR “Health Care Seeking Be-havior”[tiab] OR “Healthcare Seeking Behavior”[tiab] OR Policy[tiab] OR Poli-cies[tiab])) OR "vaccination campaign"[tiab] OR "consumer health"[tiab] OR "healthcare utilization"[tiab])) AND (“Adolescent”[Mesh] OR “Child”[Mesh] OR Ad-olescen*[tiab] OR Teens[tiab] OR Teen[tiab] OR Teenag*[tiab] OR Youth[tiab] OR Youths[tiab] OR Child[tiab] OR Children[tiab] OR Boy[tiab] OR Boys[tiab] OR Girl[tiab] OR Girls[tiab] OR “Young Man”[tiab] OR “Young Men”[tiab] OR “Young Woman”[tiab] OR “Young Women”[tiab] OR childhood[tiab])
